# Understanding the Exchange of Systemic HDL Particles Into the Brain and Vascular Cells Has Diagnostic and Therapeutic Implications for Neurodegenerative Diseases

**DOI:** 10.3389/fphys.2021.700847

**Published:** 2021-09-06

**Authors:** Juno Van Valkenburgh, Cristiana Meuret, Ashley E. Martinez, Vibha Kodancha, Victoria Solomon, Kai Chen, Hussein N. Yassine

**Affiliations:** ^1^Department of Radiology, Keck School of Medicine, University of Southern California, Los Angeles, CA, United States; ^2^Department of Medicine, Keck School of Medicine, University of Southern California, Los Angeles, CA, United States

**Keywords:** HDL, Alzheimer’s disease, APOE, ApoA-I, vascuar, imaging

## Abstract

High-density lipoproteins (HDLs) are complex, heterogenous lipoprotein particles, consisting of a large family of apolipoproteins, formed in subspecies of distinct shapes, sizes, and functions and are synthesized in both the brain and the periphery. HDL apolipoproteins are important determinants of Alzheimer’s disease (AD) pathology and vascular dementia, having both central and peripheral effects on brain amyloid-beta (Aβ) accumulation and vascular functions, however, the extent to which HDL particles (HLD-P) can exchange their protein and lipid components between the central nervous system (CNS) and the systemic circulation remains unclear. In this review, we delineate how HDL’s structure and composition enable exchange between the brain, cerebrospinal fluid (CSF) compartment, and vascular cells that ultimately affect brain amyloid metabolism and atherosclerosis. Accordingly, we then elucidate how modifications of HDL-P have diagnostic and therapeutic potential for brain vascular and neurodegenerative diseases.

## Introduction

High-density lipoprotein (HDL) particles (HDL-P) are found in the peripheral circulation as well as in the central nervous system (CNS), where they protect against disease states through a variety of physiological functions. HDL-P gain access to both parenchymal and vascular cells based on their lipid and protein composition. This allows for various degrees of exchange between blood, lymph, cerebrospinal fluid (CSF), and interstitial brain fluid compartments that is largely determined by their apolipoprotein (apo) content. Notable dissimilarities in structure and apo content distinguish periphery HDL-P from CNS HDL-P. This is likely a consequence of limited crossover between these two compartments (Mahley, 2016), however, these mechanisms have not been fully elucidated. It is pertinent to explore this gap in the literature as the manipulation of HDL-P has become a research focal point in recent years for their potential use as therapeutic and imaging agents, especially in the CNS.

The blood–brain barrier (BBB) and the blood–CSF barrier (BCSFB) compartmentalize CNS lipoprotein/apolipoprotein synthesis and metabolism separately from the periphery. HDL-P are secreted by the liver and small intestine (Timmins et al., 2005; Brunham et al., 2006), whereas CNS-derived HDL-P are generated by glial cells (Fagan et al., 1999; Vitali et al., 2014). The BBB, formed by multiple cell types, including the tightly knit endothelial cells within brain microvessels, is the greatest barrier to HDL exchange. The BCSFB, in contrast, is comprised of choroid plexus (CP) epithelial cells (McPherson et al., 2007a) that are more permeable than the BBB. This is because the junctions formed by the CP (McPherson et al., 2007a) epithelium allow for some transport of plasma proteins into the CSF (Johanson et al., 2011). In addition to these structural distinctions, the expression of transporters at the BBB and BCSFB are not the same, and the differences between their endocytic and transcytotic pathways are not clear (Strazielle and Ghersi-Egea, 2016).

While the structural, chemical, and functional properties of plasma HDL-P have been extensively studied, the properties of CSF HDL-P remain elusive due to their low abundance and high complexity (Montine et al., 1998; Yamauchi et al., 1999; Demeester et al., 2000; Koch et al., 2001; Yassine et al., 2016). Methods using gradient gel electrophoresis (Remaley et al., 2001), ion-mobility analysis (IMA), and nuclear magnetic resonance (NMR) spectroscopy have characterized three major groups of HDL-P in plasma by particle size: small (7–8.5 nm), medium (8.5–10.5 nm), and large (10.5–15 nm) (Nichols et al., 1986; Otvos et al., 1992; Jeyarajah et al., 2006; Caulfield et al., 2008). Proteomic analysis of plasma HDL isolated by density ultracentrifugation and size exclusion chromatography has identified an extensive list of over 90 proteins associated with HDL (Gordon et al., 2010; Holzer et al., 2016). In both plasma and CSF, small HDL-P are comprised of apoA-I, apoA-II, apoA-IV, apoC-I/II/III, apoD, transferrin, and other proteins, whereas apoE and apoJ are found on both smaller and larger HDL-P. Also, in both compartments, HDL-P can contain single or multiple apolipoproteins (apos) (Davidson et al., 2009) that affect their structure and function. What mainly distinguishes plasma HDL-P from CSF HDL-P is that they are enriched with apoA-I (von Zychlinski et al., 2014), while CNS HDL-P are primarily comprised of apoE (Koch et al., 2001). ApoA-I is not synthesized in the CNS, and the CSF apoA-I concentration is only 0.3% that of plasma. Furthermore, CNS-derived apoE has not been shown to cross into the periphery (Koch et al., 2017). Nevertheless, there is evidence that apoA-I is protective against CNS disease (Kawano et al., 1995; Mangaraj et al., 2016), and CNS-derived apoE has an important role in mediating amyloid-beta (Aβ) clearance (Kanekiyo et al., 2014). More broadly, enhancement of endothelial transcytosis via HDL-surface modifications has been explored as a potential drug delivery strategy to the brain (Balazs et al., 2004). Therefore, the ability to support HDL-apoA-I and/or HDL-apoE transport across the BBB may provide significant therapeutic breakthroughs in neurodegenerative diseases. This will require a deep understanding of how the BBB and BCSFB mediate the exchange of HDL-P and their components.

Not only do apos play an important role in HDL transport and component exchange, they are also involved in acute-phase response, proteolysis, immunity, Aβ clearance, and vasoprotective roles (Chait et al., 2005; Getz, 2005; Vaisar et al., 2007). Specifically, HDL-P and their components play important, protective roles against both Alzheimer’s disease (AD) and vascular dementia (VD) risk through mechanisms related to atherosclerosis, cerebral amyloid angiopathy (CAA), and inflammation (Gearing et al., 1995). However, it is not clear whether these neuroprotective properties are mediated by CNS-derived apos, through peripheral apos entering the CNS from the periphery, or via both (Remaley et al., 2001). Understanding the neuroprotective properties of apos and how they are exchanged between the periphery and CNS is crucial to understanding how HDL-P can be modified to facilitate brain delivery. In this review, we explore the known structural and functional properties of HDL-P that enable access to the brain and vascular cells, as well as their neuroprotective and vasoprotective properties. We also present evidence to support the exchange of small, lipid-poor HDL-P between the CSF and plasma compartments across the BCSFB and acknowledge that the evidence for subsequent BBB exchange is weak, and therefore requires more careful, elaborate investigations. To help bridge this gap, we present evidence that HDL-P can be modified to facilitate transport across the BBB and for imaging atherosclerosis in vessel walls.

### Mechanisms of Lipoprotein Exchange Between the Periphery and the CNS

#### Transport via the BBB

**FIGURE 1:**
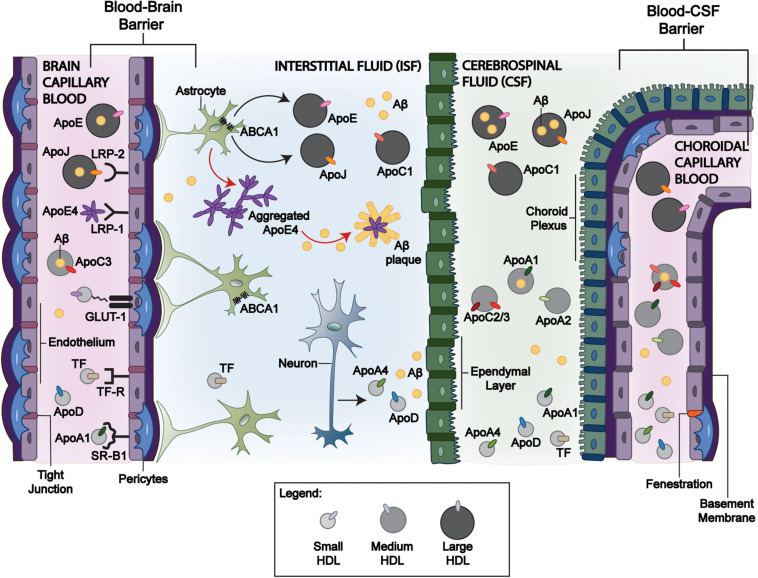
Mechanisms of HDL-P transport across blood-brain interfaces and its relevance to Aβ clearance. Circulating large apoE, apoJ, and apoC-III containing HDL-P facilitate Aβ efflux across the BBB and BCSFB through various receptor-mediated interactions and can promote Aβ efflux from the brain despite a limited BBB exchange. Of the apoE isoforms, apoE4 has the greatest affinity to LRP-1 that may allow greater BBB transport. While larger HDL-P have limited brain delivery, smaller HDL-P may exchange among the blood, CSF, and interstitial fluid compartments. ApoJ has been demonstrated to interact with LRP-2 at the BBB and BCSFB for Aβ clearance, but lipidated apoJ and apoE3 have limited brain delivery from the periphery. While peripheral apoA-I and apoA-II cross the BCSFB and are present in CSF, the size/shape that promote this exchange remains unclear. Astrocyte-derived apoJ and apoE undergo lipidation by ABCA1. In comparison to apoE2 and apoE3, apoE4 has inefficient interactions with ABCA1 (red arrow). It remains lipid-poor and is more prone to aggregation, promoting Aβ plaque formation. In contrast, the greater lipidation of apoE2 and apoE3 compared to apoE4 by ABCA1 limits the formation of Aβ plaques. LRP-1: Low-density lipoprotein receptor-related protein 1; LRP-2: Low-density lipoprotein receptor-related protein 2; ABCA1: ATP Binding Cassette Subfamily A Member 1. Blood-Brain Barrier: BBB, and Blood-CSF Barrier: BCSFB.

The BBB is formed by multiple cell types including endothelial cells, pericytes, smooth muscle cells that shields the brain from the periphery. The highly selective nature of the BBB is primarily orchestrated by receptors, which help regulate blood–CNS exchange and maintain CNS homeostasis. This results in a minimal exchange between systemic and CNS-derived HDL-P; however, small HDL and/or their components are suspected to traverse the BBB (Ladu et al., 2000; Koch et al., 2001; Wang and Eckel, 2014). Figure 1 and Table 1 show the relevant ligands and receptors of interest thought to play a role in the exchange of HDL across the BBB. ApoA-I have been shown in a more folded comformational state around small and lipid-poor HDL, giving them a discoidal form in lieu of spherically larger shape typlical of lipid-rich HDL-P. These discoildal forms were able to cross the BBB *in vitro* (Dal Magro et al., 2019), and although Martin-Nizard et al. (1989) have observed that radiolabeled HDL_3_ (small HDL, d = 1.125–1.210 g/mL) can bind the luminal membrane of cultured bovine brain capillary endothelial cells with high affinity, it remains unclear which receptors were responsible for this interaction. Furthermore, whether fully intact HDL-P do cross the BBB *in vivo* remains a point of contention. HDL are suspected to transverse the BBB via transcytosis—a process by which the HDL are internalized at the luminal surface by the endothelium, then trafficked to the basal membrane (Mehta and Malik, 2006). Some of the receptors of interest that express affinity for some relevant HDL-associated apos belong to the low-density lipoprotein (LDL) receptor superfamily. These LDL receptors, as well as scavenger receptor BI (SR-BI), are suspected to be potential mediators of HDL-transcytosis at the BBB.

**TABLE 1:** Summary of HDL-associated proteins’ source, function, and association.

Protein	Source	Cross BBB?	Cross BCSFB?	Receptor interaction	Size (Meaney et al., 2001) in Plasma	CSF/Plasma Ratio
**Transferrin**	Liver, choroid plexus, other tissues and organs	Yes	Yes	TF-R	Small HDL (Holzer et al., 2016; Kuklenyik et al., 2018)	1:150 (Memisogullari and Bakan, 2004; Mizuno et al., 2005)
**ApoA-I**	Liver, intestine	Limited BBB permeability	Yes (Stukas et al., 2014a)	Potentially involved in SR-BI mediated endocytosis of HDL at BBB [50]	Small and large HDL (Holzer et al., 2016; Kuklenyik et al., 2018)	1:700 (Koch et al., 2017)
**ApoA-II**	Liver, intestine	Unknown	Yes (Montine et al., 1998)	–	Small and large HDL (Holzer et al., 2016; Kuklenyik et al., 2018)	1:636*
**ApoC-I**	Liver, neurons, astrocytes	Some evidence (Cudaback et al., 2012)	Yes	Inhibits LRP-1, LDLR	Small and large HDL (Kuklenyik et al., 2018)	1:1,000 (Hu et al., 2020)
**ApoC-II**	Liver	–	Yes	Inhibits LRP-1, LDLR	Small and large HDL (Kuklenyik et al., 2018)	1:3,000 (Hu et al., 2020)
**ApoC-III**	Liver	Some evidence (Zhou et al., 2019)	Yes (Koch et al., 2017)	Inhibits LDLR	Small and large HDL (Kuklenyik et al., 2018), Gordts and Esko, 2018; Foley et al., 2013)	1:2,000 (Hu et al., 2020)
**ApoD**	Astrocytes, oligodendrocytes, various organs	Unknown; Hypothesized to act locally rather than in circulation (Provost et al., 1990)	Unknown	–	Small and large HDL (Holzer et al., 2016)	1:100 (Camato et al., 1989; Terrisse et al., 1998)
**ApoE**	Liver, astrocytes, macrophages	No (Elliott et al., 2010)	No (Elliott et al., 2010)	Binds LRP-1	Small and large HDL (Holzer et al., 2016; Kuklenyik et al., 2018)	1:18 (Koch et al., 2017)
**ApoJ**	Liver, astrocytes, neurons	Yes (Zlokovic et al., 1996; Bell et al., 2007; Merino-Zamorano et al., 2016)	Yes (Zlokovic et al., 1996)	Transport of soluble Aβ-apoJ complex and free apoJ across BBB and BCSFB via Megalin (LRP-2) (Zlokovic et al., 1996)	Small and large HDL (Stukas et al., 2014b)	1:950 (Koch et al., 2017)

*refers to unpublished data.

The LDL receptor family includes low-density lipoprotein receptor (LDLR), very low-density lipoprotein receptor (VLDLR), LDL receptor-related protein (LRP)-1, and LRP-2 (also known as megalin or gp330) (Herz and Bock, 2002). While most apos contain a receptor-binding domain, they exhibit different preferences for various LDL receptors. The binding affinity of apos to BBB-expressed receptors, such as the LDL receptor family, highlights important interactions that may facilitate the crossing of HDL through the BBB via receptor-mediated endocytosis and transcytosis.

LRP is expressed in brain capillary endothelium, glial cells, and neurons (Shibata et al., 2000; Herz and Bock, 2002). Its ligands of relevance for receptor-mediated endocytosis include apoE and apoJ (Bell et al., 2007; Lillis et al., 2008). LRP-1 is also expressed in smooth muscle cells (Ruzali et al., 2012), and endothelial LRP-1 transports amyloid peptides across the BBB, contributing to its clearance from the brain (Storck et al., 2016). LDLRs are of special interest as transmembrane receptors, because they are expressed on both the luminal and abluminal sides of the BBB and their affinity to apoE differs by its isoforms (Dal Magro et al., 2018): apoE4 > apoE3 >> apoE2 (Johnson et al., 2014). There are, however, limitations to apoE-HDL-mediated uptake. For example, CSF-derived apoE is not detectable in plasma (Linton et al., 1991), and how apoE isoforms differ in brain uptake is not completely understood. Liu et al. (2012) administered adenovirus encoding human apoE3 intravenously to C57BL/6J mice, and the detection of human apoE3 in the CSF was used as a surrogate measure of central availability. In mice receiving the administered apoE3 adenovirus, human apoE3 was expressed at high levels in the liver, leading to high levels of human apoE3 in mouse plasma. In CSF, however, human apoE3 levels were undetectable.

The findings from the above study may not be applicable to apoE4. In contrast to apoE4, human apoE3 has a lower affinity to murine LDL receptors that may limit its brain uptake at the BBB (Altenburg et al., 2008). Notably, Dal Magro et al. (2018) utilized the affinity of apoE4 for surfactant-stabilized nanoparticles (NPs), particularly polysorbate-80, to create an artificial protein corona that enabled the apo-decorated NP to translocate into the brain parenchyma. This process improved brain uptake three-fold compared to uncoated particles, but only at an optimal low concentration (5 μg) when the NPs did not have to compete with excess lipid-free apoE4 (Dal Magro et al., 2018). While a simple process of incubation of the NP suspension with apoE4 might prove to be a successful strategy for clinical translation, further studies need to be done to characterize the stability of the artificial protein corona, especially in circulating blood, for true translational success. In addition, a possible toxic gain-of-function effect from injecting apoE4 on several brain functions may limit its use for neurodenegnerative diseases. However, these results corroborated the hypothesis of the involvement of LDL receptors, particularly LRP-1, in receptor-mediated uptake across the BBB for apoE4-coated NPs.

ApoJ is also an LRP binding ligand and is more specific to LRP-2 (Bell et al., 2007; Lillis et al., 2008). However *in vivo*, like apoE3, HDL-apoJ appears to have limited brain delivery. Fernández-de-Retana, et al. prepared 24 and 48 nm recombinant HDL (rHDL)-apoJ NPs by assembling dipalmitoylphosphatidylcholine (DMPC) with human recombinant apoJ (rapoJ) (Fernandez-de-Retana et al., 2017). These fluorescently labeled NPs were shown to accumulate in the cranial region, especially in old transgenic mice presenting a high cerebral Aβ load, but parenchymal brain uptake was not demonstrated.

The other receptor of interest for the transcytosis of HDL is SR-BI, which is present within brain caveolae capillary endothelial cells and allows for the bi-directional movement of cholesteryl esters mediated by apoA-I (de Beer et al., 2001; Fung et al., 2017). Furthermore, apoA-I has demonstrated cerebral vascular protection and reduced AD risk (Zhou et al., 2019). Balazs et al. (2004) found evidence that part of cerebral apoA-I originates from plasma HDL and that brain capillary endothelial cells enriched with caveolae contain SR-BI receptors, which facilitated the selective uptake of HDL at the BBB. Furthermore, they demonstrated that SR-BI co-localizes with caveolin-1 (CAV-1) on brain capillary endothelial cells. In a more recent study by Fung et al. (2017), fluorescently labeled HDL was observed via high-resolution fluorescence microscopy to be internalized by SR-BI enriched within cultured human cerebral cortex microvascular endothelial cells independent of its scaffolding protein, PDZK1. Using total internal reflection fluorescence (TIRF) microscopy, HDL was further observed to be internalized by SR-BI in a manner that was independent of proximal CAV-1 and Clathrin signaling pathways. The transcytosis of HDL was, however, determined to be dependent on an unknown dynamin and cholesterol pathway. Furthermore, it was observed that HDL uptake was inhibited by the addition of 400 μg of rapoA-I (Fung et al., 2017). The antagonistic effects of rapoA-I on HDL transcytosis were determined to be reduced by 50% in the absence of SR-BI in the same study. Rohrer et al. (2009) found that adenosine triphosphate (ATP)-binding cassette transporter, ABCG1, located on bovine aortic enthodothelium, uptook HDL via transcytosis. This suggests that AB CG1 and SR-BI receptors mediate the transcytosis of HDL. It is important to acknowledge that these findings were obtained from *in vitro* studies and there has been relatively limited information on *in vivo* transfer, as discussed in sections “Transport via the BCSFB” and “Apolipoprotein E.”

### Transport via the BCSFB

The BCSFB further guards against entry into the CNS, however, studies regarding lipoprotein exchange are limited compared to the BBB. The CP, which forms the BCSFB, secretes CSF as a medium for waste removal and nutrient uptake, thus acting as an independent circulatory system in this region of the CNS. While receptors at the BCSFB are not well-defined, there is evidence that LRP-2 mediates apo transport across the BCSFB (Zlokovic et al., 1996). As shown in Figure 1 and Table 1, some plasma-derived proteins are known to traverse the BCSFB. For example, while apoA-I and apoA-II mRNAs are not expressed in brain cells, they are present in the CSF and have been associated with CNS lipoproteins (Demeester et al., 2000). Stukas et al. (2014a) identified that the majority of intravenously injected lipid-free apoA-I was found in the CP *in vivo*. Like apoA-I, peripheral apoA-II is suspected to cross the BCSFB through the CP. In AD and control brain tissue, apoA-II immunoreactivity was observed in the cytoplasm of CP epithelium and within blood vessels consistent with a pattern for transport across the BCSFB (Montine et al., 1998). However, it is unclear if apoA-I-containing particles aid the transport of other plasma proteins into CSF or if CNS-expressed receptors/transporters directly facilitate the transcytosis of circulating proteins (Montine et al., 1998; Stukas et al., 2014a; Koch et al., 2017). What is clear is the lipidation and subsequent shape of HDL-P are in constant flux at the BCSFB.

Exchangeable apos existing on small, lipid-poor HDL-P or circulating in their lipid-free form are suspected to become lipidated in the CSF compartment following entry from the periphery. Though not as efficient as apoA-I, exchangeable apos, such as apoA-II, apoA-IV, apoC-I, apoC-II, apoC-III, and apoE, have been identified as suitable activators of ATP-binding cassette subfamily A member 1 (ABCA1)—a transport protein responable for mediating the efflux of cholesterol and phospholipids to lipid-poor/free apos (Remaley et al., 2001; Pearson et al., 2004). Fujiyoshi et al. (2007) detected ABCA1 and ABCG1 mRNAs and proteins in isolated rat CP. Additionally, they found that both ABCA1 and ABCG1 on CP epithelium are involved in the transfer of cholesterol and lipids to lipid-poor apos and lipoproteins in CSF (Cavelier et al., 2006; Fujiyoshi et al., 2007). This suggests that the lipidation of delipidated and/or lipid-poor apos within the CP occurs in lieu of HDL transport across the BCSFB from the periphery. This hypothesis is supported by associations among plasma and CSF apos’ concentrations (Koch et al., 2017; Hu et al., 2020). The process in which lipid-poor HDL-P or lipid-free apos (made of apoA-I, apoA-II, apoA-IV, apoCs, and apoE) originating in the periphery become lipidated by CNS-expressed transporters following transport across the BCSFB likely affects brain Aβ accumulation and is discussed in section “Effect of HDL Proteins on CNS Aβ Accumulation and Related Pathology.”

### Surface Modifications to sHDL to Enhance Brain and Cellular Access

There are several HDL modifications shown to enhance their BBB transport. Transferrin is an iron-binding protein that is well known for its antioxidant capacity and ability to traverse both the BBB and BCSFB (Table 1). In plasma, transferrin has been shown to associate with apoA-I containing HDL-P isolated by selected affinity immunosorption (Kunitake et al., 1992) and denser HDL-P isolated by ultracentrifugation (McPherson et al., 2007b). With its receptors expressed on brain capillary endothelial cells (Visser et al., 2004; Johnsen and Moos, 2016), NPs modified with transferrin (Tf) are being extensively studied for drug delivery as potential treatments for brain cancers and several neurodegenerative diseases (Wiley et al., 2013; Johnsen et al., 2019; Ullman et al., 2020). Tf receptor ligands have also been incorporated into NPs in order to further facilitate transmission across the BBB. Clark and Davis (2015) demonstrated that 80 nm gold NPs that were bound to Tf by an acid-cleavable linker were better able to facilitate receptor-mediated transcytosis (RMT) and avoid BBB endothelium retention by shedding surface Tf upon acidification during transcytosis. The targeted NPs showed greater permeability across BBB models *in vitro* and entered mouse brain parenchyma in greater amounts when compared to NPs with non-cleavable Tf. Additionally, Cui et al. (2018) constructed a dual-modified HDL containing T7, a transferrin receptor ligand, and dA7R, a peptide used for its glioma-homing property, that displayed higher glioma localization than that of single ligand-modified HDL. Both these findings demonstrated that incorporating Tf-like ligands into the modification of natural HDLs could prove to be a more successful methodology for the delivery of therapeutic agents across the BBB in lieu of apo-specific receptors.

dos Santos Rodrigues et al. (2019) proposed the enhanced brain targeting and gene delivery of dual-modified (Penetatrin-Transferrin) liposomes encapsulating plasmid *APOE2* as a new gene-targeting therapeutic approach for the treatment of AD. The liposomes were surface modified with Tf, similar to previously mentioned studies, but also incorporated the conjugation of DSPE-PEG-liposomes to Penetratin (Vaisar et al., 2007)—a cell-penetrating peptide implicated with a critical enhancement of the translocation of associated cargo, such as Pen-associated liposomes—across cellular membranes, such as the BBB. A singular intravenous injection of the dual-modified liposomes loaded with plasmid *APOE2* increased apoE expression in the brain of these mice models and demonstrated successful translocation across *in vitro* triple co-culture BBB models. This study provides Tf-Pen modified liposomes as an effective method for brain delivery of plasmid *APOE2*, which has shown neuroprotective properties and a greater binding affinity to Aβ.

Indeed, one of the largest concerns in the clinical translation and the general success of synthetic HDLs is the low permeability and poor targeting property of HDLs across the BBB. Therefore, the study by dos Santos Rodrigues et al. (2019) highlights the efficacy of how the dual presence of the Tf ligand, in mediating transport across the BBB through RME, and the Pen peptide, in enhancing liposome internalization into cells, ultimately overcomes receptor saturation and promotes transfection in successfully transported HDL. Additionally, the surface modification also increased the stability of the liposome. The use of DSPE-PEG phospholipids minimized protein interaction and recognition by macrophage, while also reducing NP clearance through prolonged circulation. The plasmid DNA complexed to chitosan improved transfection by sterically hindering nucleases from degrading the nucleic acids and was also optimized to ensure nucleic acids released at target sites. Lastly, the low hemolytic activity of the liposomes at low phospholipid concentrations indicated blood compatibility safe for intravenous injection. This study demonstrated an effective method of apoE2 brain delivery that has potential for AD treatment and clinical translation.

An additional alternative method for enhanced HDL penetration across the BBB is *via* specific glycosylation. Zhou et al. (2020) developed a glycosylated siRNA NP delivery system (Gal-NP@siRNA) with “triple interaction” stabilization that specifically silenced BACE1 preemptively to decrease Aβ levels in a transgenic AD mouse model. To facilitate the transportation of the nanomedicine across the BBB, the glycosylated nano-delivery system hacked the recycling of the glycemia-controlled glucose transporter 1 (Glut1) receptor, which resulted in the movement of Glut1 from the luminal to the abluminal side of the BBB after treatment with Gal-NP@siRNA due to glucose replenishment. The “triple interaction”, more specifically, the electrostatic and hydrogen bonding interaction of the guanidinium-phosphate bridge and the fluorine-mediated hydrophobic interaction between the siRNA and the galactose-modified polymer mixture, improved the biophysiological protection of the siRNA and the stability of the NPs in blood circulation. Gal-NP@siBACE1 successfully decreased BACE1 expression for at least 3 days, consequently reducing Aβ plaque levels and suppressing phosphorylated tau protein levels. This further resulted in regeneration of impaired myelin, suggesting a clearance of by-products due to biocompatibility, and contributed to the restoration of cognitive function in transgenic AD mice models. The long-term effects of these injections on Aβ levels are not clear, however. Due to the stability, ease of formulation, and successful BBB penetration (among other factors), the Gal-NP@siBACE1 model demonstrated promising potential for clinical translation, and the study provides support for the use of RNA interference therapy for AD.

### The Effect of HDL Proteins on CNS Aβ Accumulation and Related Pathology

There is a large body of research demonstrating that various apos play a large role in attenuating toxic Aβ pathology in the brain. The main apos of interest are apoE, apoJ, apoA-I, and apoD. The HDL components tested for brain delivery and impact on Aβ accumulation are summarized in Tables 1 and 2, and illustrated in Figure 1.

**TABLE 2:** HDL-proteins for brain delivery and impact on Aβ accumulation.

Protein	Lipid	Cross BBB?	Size (nm)	Mechanism of delivery	Model*: in vitro*, *in vivo*	AD effect	References
ApoE	DMPC	Yes	21–27	RMT	microglial cells, primary astrocytes, liver cells	High Aβ binding affinity, accelerated Aβ degradation via lysosomal transport, rescued memory deficit	Song et al., 2014
					AD animal model (SAMP8, SAMR1)		
ApoE	DMPC	Yes	27	RMT	Mouse brain endothelial cell (bEnd.3) line, microglia (BV2) cell line	Enhanced Aβ binding affinity decreased amyloid deposition, rescued memory deficit	Song et al., 2016
					AD mouse model (SAMP8)		
ApoE	DMPC	Yes	26-27	LDLR-mediated transcytosis	Mouse brain endothelial cell (bEnd.3) line, microglia (BV2) cell line	Enhanced Aβ binding affinity, reduced Aβ deposition, attenuated neurological damage, rescued memory deficits	Song et al., 2018
					AD animal model (SAMP8, SAMR1)		
ApoE	cetyl palmitate	Yes	211	LDLR (LRP-1) mediated uptake	human cerebral microvascular endothelial (hCMEC/D3) cells	none (not primarily discussed)	Dal Magro et al., 2018
					Male BALB/c mice		
rApoJ	DMPC	Yes	24,48	not discussed	Mouse J774A.1 macrophage-like cells	Improved *in vitro* cholesterol efflux abilities, prevented Aβ fibrillization	Fernandez-de-Retana et al., 2017
					Transgenic mouse model with high cerebral Aβ load (APP23)		
ApoA-I	none	Yes	N/A	specific cellular mediated transcytosis	Human choroid plexus epithelial cells brain microvascular endothelial cells	None (not primarily discussed)	Stukas et al., 2014a
					C57Bl/6 mouse model		
ApoA-I	none	Yes	N/A	clathrin independent cholesterol-mediated endocytosis	hCMEC/D3 endothelial cell monolayers	None (not primarily discussed)	Zhou et al., 2019
					Wild-type male rat models		
4F ApoA-I	none	Yes	N/A	RMT	BBB endothelial cell monolayers (hCMEC/D3)	Reductions in brain Aβ burden	Swaminathan et al., 2020
					B6SJLF1/J wild type AD mice models		

*RMT, receptor-mediated transcytosis; RME, receptor-mediated endocytosis; LDLR, low-density lipoprotein receptor.*

### Apolipoprotein E

Apolipoprotein E (ApoE) is highly expressed in the CNS primarily by astrocytes (Elshourbagy et al., 1985; Mahley, 1988), but to a lesser extent in microglia (Butovsky et al., 2014), pericytes (Blanchard et al., 2020), and stressed neurons (Mahley and Huang, 2012). CNS-derived apoE is known to transfer phospholipids and cholesterol via interaction with ABCA1 and ABCG1, and promote axonal growth via interactions with the LDLRs (Fagan et al., 1998; Wahrle et al., 2004; Kim et al., 2007). ApoE lipidation via its interaction with ABCA1 has important implications toward Aβ clearance (Wahrle et al., 2004, 2005, 2008; Hirsch-Reinshagen et al., 2005; Koldamova et al., 2005; Fitz et al., 2012). Lipid-poor apoE aggregates (Hatters et al., 2006) are central to the formation of Aβ plaques, and are exacerbated by the *APOE4* genotype (Liao et al., 2018). The lipidation of apoE by ABCA1 agonists attenuates this aggregation (Rawat et al., 2019). We have shown that preserving ABCA1 function using the ABCA1 agonist, CS-6253, enhanced the ability of astrocytes to lipidate apoE4 and degrade Aβ peptides. This suggests that the transport of apos into the brain, which can activate and stabilize ABCA1, offers a therapeutic approach to limit apoE aggregation and Aβ plaque formation as illustrated in Figure 1.

ApoE-rHDL has been presented as a novel nanomedicine for the treatment of AD. Song et al. (2014) utilized 21–27 nm apoE3-rHDL nanostructures in an attempt to lower brain Aβ accumulation in an aging mouse model. The injected particles likely interact with ABCA1 and ABCG1 *in vivo* that modifies the shape of rHDL after injection. ApoE-rHDL injections had limited (0.4% ID/g) access to the CNS but demonstrated lower Aβ accumulation in these aging mouse models (Song et al., 2014). These effects were amplified by adding Mangostin, a model drug that accelerates Aβ degradation, to apoE containing HDL-P, and demonstrated enhanced degradation of Aβ and improved memory deficits (Song et al., 2016). In a more recent study, Song et al. (2018) examined the effects of rHDL’s shape on its brain delivery, Aβ degradation, and anti-AD efficacy by comparing 27 nm spherical and discoidal apoE3-NPs nanocarriers. Spherical NPs, relative to discoidal particles, exerted the best effect due to superior brain distribution after intravenous administration, powerfully reduced Aβ deposition, decreased microglia activation, attenuated neurological damage, and rescued memory deficits in the same aging model. Notably, the NP size (27 nm) is larger than that of HDL-P (7–15 nm). It is not clear why these larger spherical particles had greater brain penetration, considering in previous studies brain delivery of apoE3-rHDL was largely unsuccessful in mouse models.

### Apolipoprotein J

Apolipoprotein J (ApoJ), also known as clusterin, is an Aβ chaperone, as previously noted. It is typically associated with HDL in plasma and is a major component of CSF, wherein it is found on very dense, lipid-poor HDL-like and large HDL-P secreted by neurons and astrocytes (de Silva et al., 1990a,b; Suzuki et al., 2002). Cole et al. demonstrated that plasma-isolated apoJ lipidated with DMPC, as well as plasma-derived HDL, mediated Aβ degradation in rat microglia *in vitro* (Cole et al., 1999). These injections reduced Aβ accumulation in a similar manner to the rHDL-apoE3, possibly by facilitating Aβ efflux from the brain at the BBB. Whereas apoE, particularly apoE4, has a preference for binding LRP-1, ApoJ primarily interacts with the megalin/LRP-2 (Figure 1), where it facilitates Aβ clearance across the BBB and BCSFB (Zlokovic et al., 1996; Bell et al., 2007; Verghese et al., 2013). Indeed, Zlokovic et al. (1996) found that apoJ demonstrated a higher permeability–surface area product (Pearson et al., 2004) than apoE, and Bell et al. (2007) found Aβ42 complexed with apoJ was cleared 83% faster in murine models than Aβ42 alone.

### Apolipoprotein A-I

Clinical studies have shown that lower plasma HDL cholesterol (HDL-C) and apoA-I concentrations are associated with increased severity of AD (Merched et al., 2000; Zuin et al., 2021). An amyloid PET brain imaging study demonstrated an association between low levels of serum HDL-C and greater cerebral amyloidosis (Reed et al., 2014). Similarly, greater serum HDL-C has been shown to be associated with greater cognitive function (Bates et al., 2017). These associations suggest a protective role for apoA-I on cognition and brain amyloidosis. While there is some evidence that plasma-derived/liver apoA-I expression may not alter parenchymal Aβ deposition, several studies reported that apoA-I attenuates cerebral Aβ angiopathy, reduces neuroinflammation, and preserves cognitive function. Specifically, Lewis et al. (2010) reported transgenic (TG) mice that overexpressed amyloid-β precursor protein (APP) and presenilin 1 (PS1), but without apoA-I expression, exhibited learning and memory deficits, higher levels of cerebral Aβ angiopathy, and Aβ-induced inflammation compared to APP/PS1/apoA-I-overexpressing mice. However, no significant differences in brain Aβ depositions between these two groups were detected (Lewis et al., 2010). Lefterov et al. demonstrated that both lipidated and non-lipidated apoA-I attenuated Aβ42 aggregation and toxicity in primary brain cells, and further showed that while apoA-I deficiency did not affect APP processing and soluble/insoluble brain parenchyma Aβ levels, 12-month-old APP/PS1 mice lacking apoA-I had higher insoluble Aβ levels in cerebral blood vessels and memory deficiencies (Lefterov et al., 2010). While these results were reaffirmed by a recent study by Robert et al. (2020), the underlying mechanisms remained unclear.

Though Contu et al. (2019) attempted to elucidate these mechanisms in apoA-I deficient AD mice models, their study obtained contrary results using the TG2576 model. This model also uses an APP mutant which expresses high Aβ levels so that by 11–13 months, these mice exhibit pathological vascular amyloid and parenchymal Aβ plaques. Furthermore, APP/PS1 mice have a different age of Aβ-pathology onset (6–9 months) than the TG2576 mice (9–12 months). Contu et al. (2019) had reported that following direct injection of Aβ into the hippocampal region, apoA-I deficient mice had higher perivascular Aβ drainage and less parenchymal and vascular Aβ pathology than the controls. They further observed that levels of apo-associated transporters, like AB CA1, LRP-2, and LRP-1, had not increased, nor had apoD and apoE levels. Clusterin/apo-J plasma and cortex levels were, however, higher in apoA-I deficient mice. These studies highlight not only the nuances in using different AD mouse models to study apo-related effects on Aβ pathology, but also how dyslipidemia affects certain apos’ neuroprotective effects.

### Apolipoprotein D

In contrast to other apos that are synthesized primarily in the liver and intestine, apolipoprotein D (ApoD) synthesis extends to the CNS and other peripheral organs such as the adrenal glands, kidneys, and pancreas (Drayna et al., 1986). ApoD is associated with lipoprotein subclasses in human CSF (Borghini et al., 1995; Koch et al., 2001). As a member of the lipocalin family, apoD shares little structural homology with other apos. As a result, apoD is unable to support the synthesis of nascent HDL (Flower et al., 2000; Rassart et al., 2000; Eichinger et al., 2007). However, the hydrophobic surface properties of apoD can explain its association with HDL-P and its ability to interact with lipid membranes (Eichinger et al., 2007). Known for its potent antioxidant properties, apoD is suspected to play a role in antioxidation in the brain. In an apoD knockout mouse model, increased sensitivity to oxidative stress was observed along with compromised nervous system function and decreased life expectancy, whereas overexpression of apoD in this mouse model resulted in increased resistance against oxidative stress (Ganfornina et al., 2008).

Along with apoE and apoJ, apoD is expressed at high levels in the prefrontal cortex, and apoD expression increases 5-to-10-fold during normal aging (Kim et al., 2009; Elliott et al., 2010). In AD patients, apoD expression is increased in the hippocampus, entorhinal cortex, pyramidal cells, and CSF when compared to controls (Terrisse et al., 1998; Kalman et al., 2000; Rassart et al., 2000; Belloir et al., 2001). Regarding Aβ pathology, dual-immunolabeling of temporal cortex tissue in control and AD individuals revealed that 63% of Aβ plaques co-localized with apoD, and increased immunoreactivity was observed in glial cells and cerebral vasculature (Desai et al., 2005). While the role of apoD in AD pathogenesis is unclear, it is possible that apoD partakes in Aβ-related pathology and/or the oxidative stress response in neurodegeneration.

### Apo-Peptides

More recently, Swaminathan et al. (2020) looked at a therapeutic alternative to apoA-I-HDL-P due to their low permeability across the BBB in the form of 4F, an 18 amino acid apoA-I mimetic peptide, by examining the permeability-surface area product at the BBB and its effects on ^125^I-Aβ trafficking from brain-to-blood and blood-to-brain. They demonstrated a ∼1,000-fold higher permeability for ^125^I-4F compared to those determined for ^125^I-apoA-I. Treatment with 4F also increased the abluminal-to-luminal flux and decreased the luminal-to-abluminal flux of ^125^I-Aβ42 across BBB endothelial cell monolayers *in vitro*, as well as decreased the endothelial accumulation of fluorescein-labeled Aβ42. These results provided a mechanistic interpretation for the reductions in brain Aβ burden reported in AD mice after oral 4F administration, which represents a novel strategy for treating AD and CAA (Swaminathan et al., 2020). Our studies indicate that the ABCA1 agonist CS-6253 (modeled after the C-terminus of apoE) reduces brain apoE aggregation (Rawat et al., 2019), and attenuates AD pathology (Boehm-Cagan et al., 2016), although the brain delivery of CS-6253 is not clear. It is plausible that ABCA1 agonist peptides activate the peripheral sink of Aβ by promoting the formation of Aβ-binding lipoprotein particles into circulation.

### Alternative Mechanisms for How Apo-HDLs Affect CNS Aβ Accumulation

Concordantly, increased apo-HDL penetration may not solely explain its neuroprotective benefits. The Peripheral-Sink Hypothesis proposes that Aβ-binding ligands in the periphery can promote CNS Aβ efflux by sequestering Aβ into the peripheral circulatory system. Indeed, increasing peripheral Aβ antibodies has been shown to increase Aβ efflux (Lemere et al., 2003; Deane et al., 2009) through LRP-1 (Kang et al., 2000; Shibata et al., 2000).

Aβ sequestration into the periphery may begin with apoJ as a chaperone from the ISF, as shown in Figure 1. Bell et al. (2007) demonstrated that apoJ cleared [^125^I]-labeled Aβ40 and Aβ42 across the BBB via LRP-2. They furthermore showed that Aβ42–apoJ, compared to Aβ42 alone, crossed the BBB at an increased rate of 83%. It is possible that Aβ then crosses into the CSF following an influx of plasma-derived apoA-I. Human apoA-I overexpression in AβPP/PS1 transgenic mice was demonstrated to increase plasma HDL levels and preserve cognitive function via Aβ sequestration (Paula-Lima et al., 2009; Lewis et al., 2010). Robert, et al. demonstrated that apoE and apoA-I on HDL promoted Aβ transport across bioengineered human cerebral blood vessels, although in one of these studies recombinant apoE was injected into the “brain side” of the engineered vessel (Robert et al., 2017, 2020). Aβ preferentially binds to HDL apoA-I, but secondarily to VLDL apoE and apoC-III (Bell et al., 2012). Once in the CSF, Aβ may then cross into the periphery with an increase in plasma apoE and apoC-III concentrations. Shih et al. (2014) revealed apoC-III is an Aβ binding protein in the periphery. Interestingly, apoE4 carriers were shown to have lower peripheral apoE and apoC-III levels (Olivieri et al., 2007). These studies are indicative of higher plasma apoA-I, apoE, and apoC-III may preserve cognitive function in AD models (Lewis et al., 2010; Shih et al., 2014; Wang et al., 2019), and further demonstrate that factors driving the exchange of HDL and its components between the periphery and CNS require further elucidation.

### sHDL to Image Vascular Atherosclerosis and Its Relevance to the Brain

**FIGURE 2:**
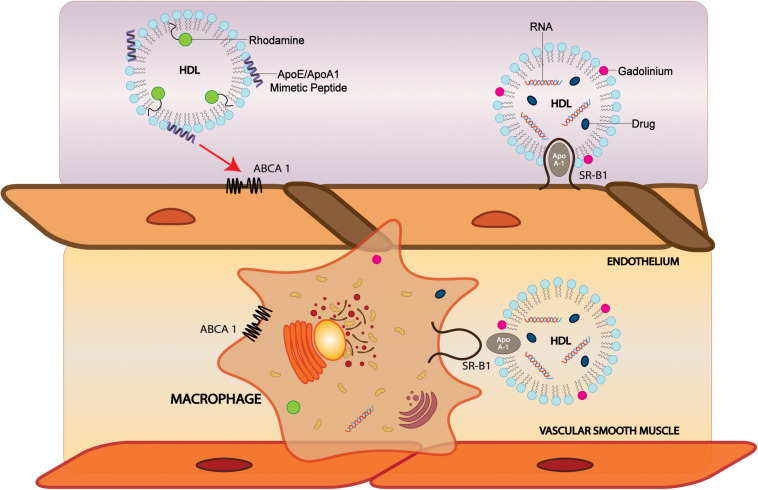
Modification of sHDL particles can facilitate brain, vascular cell, and macrophage delivery. HDL-like NPs labeled with phospholipid-containing dyes such as rhodamine and probes conjugated with CS-6253, an ABCA1 agonist, can target ABCA1 on endothelial cells and macrophages to deliver cargo. Incorporation of MRI contrast agents such as gadolinium into HDL can increase signal. In SR-BI positive cells, sHDLs comprised of apoA-I can bind SR-BI and are taken up by the cell where cargo is delivered. ABCA1, ATP Binding Cassette Subfamily A Member 1; SR-BI, Scavenger receptor, class B type 1.

HDL-apos have additional important roles in imaging vascular atherosclerosis that is mechanistically linked to both AD and vascular dementia, and other CNS diseases (Chui et al., 2012). In AD mouse models, genetic apoA-I deficiency showed exacerbated memory deficits and increased CAA (Lefterov et al., 2010). Since atherosclerosis and vascular Aβ accumulation are mechanistically linked (Gupta and Iadecola, 2015), we discuss here properties that enable HDL-P to access blood vessels that could be pursued for vascular imaging of brain atherosclerosis. The mechanisms of HDL access to the vascular component are illustrated in Figure 2.

### sHDL MRI Contrast Agents

Because of the association of vascular pathology with AD-linked biomarkers (Gupta and Iadecola, 2015) and dementia, the ability to image vasculature could be valuable for monitoring the progression of neurodegenerative diseases. HDL may be modified to include a variety of materials that generate contrast for medical imaging. These materials include radioactive or paramagnetic elements, fluorophores, and nanocrystals. One application for using HDL as an imaging agent was directed at macrophages to image atherosclerotic plaques using MRI. The first HDL contrast agent for MRI was developed by Frias et al. (2004, 2006). They reconstituted HDL with gadolinium-chelated lipids and a fluorescent dye with apoA-I and a cholesteryl ester core to make spherical particles, and later made similar Gd-HDL-P with discoidal morphology (Frias et al., 2004, 2006). The agent was administered to apoE KO mice, and MR imaging showed a significant increase in accumulation in abdominal aortas of the mice, which was confirmed to be due to the accumulation of the particles in macrophages and atherosclerotic plaques by *ex vivo* fluorescence imaging. They then improved the imaging capabilities of the particles by introducing novel gadolinium-chelating lipids, which allow for two water molecules to bind to the gadolinium instead of one, increasing the longitudinal relaxivity by a factor of four (Briley-Saebo et al., 2009). Signals can also be increased by incorporating multiple gadolinium-lipids (Gd-lipids) into each particle (Ramos-Cabrer et al., 2016). Gadolinium has also been incorporated into HDL via conjugation to apoA-I (Sriram et al., 2011; Lagerstedt et al., 2013), cholesterol (Rui et al., 2012), and to long-chain hydrophobic molecules that intercalate into the lipid coat (Carney et al., 2015). While gadolinium is the most popular element for HDL MRI contrast agents, a europium contrast agent has also been developed for use in paramagnetic chemical exchange saturation transfer (PARACEST), a highly sensitive type of MRI (Wang et al., 2015). Notably, the application of these imaging methods to either AD mice models or to human brains is still lacking and has great potential to help delineate the interaction of atherosclerosis with AD and vascular dementia pathology.

HDL MRI contrast agents have also been made with Apo-mimetic peptides. Synthetic peptides can confer advantages over native apoA-I in that they can be easily chemically synthesized and modified, and they do not require plasma-derived products and thus are safer for use in patients without extensive purification. Alpha-helical peptides from truncated apoA-I labeled with gadolinium have shown improved macrophage and plaque targeting *in vivo* compared to full-length apoA-I (Sigalov, 2014), and increasing the Gd-loading per particle increased the atherosclerotic wall/muscle normalized enhancement ratio by 160% (Shen et al., 2015). Cormode et al. developed the Gd-HDLs, prepared particles using 18A and 37pA, which are 18 and 37-residue amphipathic helical peptides mimicking apoA-I function (Cormode et al., 2008a, 2009). Both types of particles had high cholesterol efflux and were selectively taken up in macrophage cells over smooth muscle cells. The signal decreased by competition with unlabeled HDL, showing that the uptake is receptor dependent. Further studies by the same group used P2A2, a peptide derived from the LDL receptor domain of apoE, for Gd- labeled HDL-P (Chen et al., 2008). In macrophages, the uptake signal measured by MRI and fluorescence was higher than Gd-HDL made with full-length apoA-I, and the MRI signal was also higher than the signal from Gd-HDL in the aortas of apoE KO mice.

### HDL as a Mechanism to Reroute Contrast Agents

HDL is directed to its natural targets by apoA-I binding. Because HDL can be reconstituted or conjugated with other peptides and proteins, it can be redirected. MRI contrast agents based on HDL have been delivered to targets other than the natural targets that are overexpressed in cardiovascular disease or cancer. HDL was conjugated with collagen-specific EP3533 peptides (EP3533-HDL) to monitor atherosclerotic plaque regression by MRI in a Reversa mouse model. Collagen, which is a marker of plaque stability, can be used as a target to distinguish between collagen and other components of the extracellular matrix. The NPs were labeled with gadolinium and administered to the atherosclerotic mice, whose regression was induced with a genetic switch. At 28 days after induction of plaque regression, there was a significant increase in MR signal from EP3533-HDL which corresponded to the increase of collagen in the plaques. *Ex vivo* confocal microscopy of aortic sections showed HDL colocalized with macrophages and not collagen, while EP3533-HDL colocalized with collagen and not macrophages (Chen et al., 2013).

### SR-BI Uptake Mechanism for Direct Cytosolic Delivery

HDL and HDL-like NPs have been labeled with phospholipids containing fluorescent dyes such as rhodamine (Cormode et al., 2008b, 2009) and nitrobenzoxadiazole (Frias et al., 2004, 2006), lipophilic dyes or fluorescent nanocrystals loaded into the core of the NPs (Cormode et al., 2008b; Chen et al., 2014), and with probes conjugated to the lipoprotein (Kim et al., 2014) or peptide components (Zhang et al., 2009). Because fluorescence imaging has the spatial resolution to distinguish cellular localization, it is a useful modality for elucidating the mechanism of NP interaction with cells. By labeling different NP components (core, lipid layer, protein/peptide), the localization of the components can be imaged by fluorescence.

To investigate the mechanism of drug delivery of HDL NPs, Zhang et al. (2009) developed multi-labeled nanocarriers comprised of a DiR-BOA core and an apoA-I mimetic peptide with a phospholipid coat, with fluorescein labeling on either the peptide or phospholipids. In SR-BI-positive cells, the cargo dye signal was observed in the cytosol and did not colocalize with LysoTracker, whereas the peptide and phospholipid signals were retained on the cell surface. Since SR-BI facilitates the uptake of lipids from hydrophobic cores of lipoproteins, and lipid-soluble molecules, it is not surprising that dyes and hydrophobic drugs carried to the cell by HDL can be transported into the cytosol by SR-BI. These findings suggest that via SR-BI, HDL nanocarriers are viable direct-cytosolic delivery systems for hydrophobic drugs that are prone to lysosomal degradation.

### PET Imaging of Atherosclerosis With sHDL

Coupling PET tracers to HDL allows the sensitive tracers to access vascular tissues so they can be imaged with high specificity. Pérez-Medina and coworkers developed macrophage-targeting rHDLs radiolabeled with ^89^Zr on ApoA-I (^89^Zr-A1-HDL) or phospholipids (^89^Zr-PL-HDL) for imaging atherosclerosis in murine, rabbit, and porcine models (Pérez-Medina et al., 2016). Biodistribution studies showed uptake in atherosclerotic tissues as well as kidneys, liver, spleen, and bone marrow. PET/CT of rabbit aortas with atherosclerotic lesions showed higher uptake of ^89^Zr-PL-HDL than the control (0.31 ± 0.10 vs 0.16 ± 0.03 g/mL, *p* < 0.05). In pigs, atherosclerotic femoral arteries had a high accumulation of ^89^Zr-PL-HDL at 48 h post-injection. The ability of these particles to preferentially target macrophages and plaques makes them promising imaging agents for multiple diseases.

CER-001, a pre-β-HDL mimetic containing human recombinant apoA-I and phospholipids, has also been used for imaging atherosclerosis. Zheng et al. (2016) labeled the apoA-I component of CER-001 with ^89^Zr and performed serial PET/CT imaging in human patients. Patients with atherosclerotic carotid artery disease (*n* = 8) were give unlabeled CER-001 (3 mg/kg) with ^89^Zr-CER-001 (10 mg) in a 1 h infusion. PET/CT images showed carotid artery uptake of ^89^Zr-CER-001, expressed as target-to-background ratio (TBR_*max*_), was significantly increased at 24 h after infusion compared to initial scans 10 min after infusion (1.14 vs. 0.98; *p* < 0.001) and remained increased at 48 h (1.12, *p* = 0.007). TBR_*max*_ in plaque was 1.18, which is significantly higher than non-plaque areas (1.05, *p* < 0.001).

^18^F-Fluorodeoxyglucose (^18^F-FDG) accumulates in inflammatory cells associated with atherosclerotic plaques, but is non-specific and thus not ideal for imaging of atherosclerosis. Yong-Sang and coworkers synthesized ^68^Ga-labeled HDL-P labeled on the phospholipid and compared them to ^18^F-FDG as PET probes for imaging atherosclerotic plaques (Yong-Sang et al., 2019). The Saku group developed a PET probe to image atherosclerosis based on a 24-amino acid apoA-I mimetic peptide known as Fukuoka University apoA-I Mimetic Peptide (FAMP), which promotes macrophage reverse cholesterol transport (RCT) in a cholesterol-fed mouse model (Kawachi et al., 2013). FAMP was modified with DOTA and labeled with ^68^Ga, then injected into the myocardial infarction animal model, Watanabe heritable hyperlipidemic rabbits (WHHL-MI). Atherosclerotic plaques and aortic atherosclerotic plaques in WHHL-MI rabbits showed high uptake of ^68^Ga-DOTA-FAMP compared to wild-type rabbits (Kawachi, 2015).

## Conclusion and Future Perspectives

HDL-apos are important determinants of AD pathology and VD having both central and peripheral effects on brain Aβ accumulation and vascular functions. Although the extent to which HDL-P can exchange their protein and lipid components between the CNS and the systemic circulation is still not clear, HDL-P offer untapped therapeutic potential for vascular and neurodegenerative diseases through the following mechanisms that warrant additional examination:

1. Small HDL-P appear to gain access into the brain compartment *in vitro*, but further studies are required to identify their transport *in vivo* and the small HDL components that render these BBB and BCSF transport properties, including apo and lipid composition, size, and shape.
2. Lipid-poor HDL-P entering the brain or CSF are lipidated in the brain via interactions with ABCA1/ABCG1. This process may allow for the exchange of brain lipids with peripheral lipoproteins and has important implications for Aβ production and its clearance from the brain.
3. Modifying HDL-P (e.g., the addition of Tf peptide) can enhance its brain delivery via the Tf receptors at the BBB, but applications for modifying HDL to enhance its brain delivery in neurodegenerative diseases are still lacking.
4. Even without access into the brain, some lipoproteins in the circulation can sink Aβ from the brain, a process that involves lipoprotein-Aβ binding in the circulation. This point is important for developing brain lipoprotein therapeutics without the prerequisite of crossing into the brain as a drug development milestone.
5. Since atherosclerosis is involved in the pathogenesis of brain amyloidosis and VD, imaging atherosclerosis in the brain via HDL NPs can delineate mechanisms of dementia in both parenchymal and vascular amyloidopathies, and guide drug treatments that have dual effects, ameliorating both atherosclerosis and vascular amyloid deposition.

## Author Contributions

HY and KC designed the review. JV, CM, AM, VK, and VS wrote the manuscript. All authors reviewed the manuscript.

## Conflict of Interest

The authors declare that the research was conducted in the absence of any commercial or financial relationships that could be construed as a potential conflict of interest.

## Publisher’s Note

All claims expressed in this article are solely those of the authors and do not necessarily represent those of their affiliated organizations, or those of the publisher, the editors and the reviewers. Any product that may be evaluated in this article, or claim that may be made by its manufacturer, is not guaranteed or endorsed by the publisher.
